# The Strychnine Treatment of Pulmonary Consumption

**Published:** 1895-05

**Authors:** 


### THE STRYCHNINE TREATMENT OF PULMONARY CONSUMPTION.

NEXT to rest and food, strychnine in large doses is the most important agent in the treatment of pulmonary consumption. Begin with 1-32 of a grain and gradually increase to 1-16, 1-10 or 1-6 of a grain or even larger doses, given four times a day. According to the author, it does not produce albuminuria or diabetes as is generally supposed. It alleviates the loss of appetite, the vomiting, the constipation, the nervousness and sleeplessness, the pain in the chest, the cough and expectoration, the dyspnea, the weakness of the heart, and acts as a blood-builder in an eminent degree. Its usefulness rests, of course, on its influence over the nervous system, and is another link in the chain of evidence which shows that in the great majority of cases pulmonary consumption is the direct result of primary disease of the pulmonary nerve supply.—*Thomas J. Mays, in College and Clinical Record.*

---

THE disposition to seize every opportunity to dose the children was beautifully illustrated by a physician's experience not long ago. He was hastily called to see a youngster of three years, who had fallen out of a window about four feet to the brick pavement below, landing on his head. When he reached the scene, the mother, with pardonable pride in her presence of mind and domestic resources, exclaimed: "Oh, doctor, I knew that something ought to be done at once, so I gave him two teaspoonfuls of castor oil."—*American Med. Compend.*

